# Huangqin Tea Total Flavonoids–Gut Microbiota Interactions: Based on Metabolome and Microbiome Analysis

**DOI:** 10.3390/foods12244410

**Published:** 2023-12-07

**Authors:** Yaping Zheng, Kailin Yang, Jie Shen, Xiangdong Chen, Chunnian He, Peigen Xiao

**Affiliations:** 1 Institute of Medicinal Plant Development, Chinese Academy of Medical Sciences, Peking Union Medical College, Beijing 100193, China; zyp0316@163.com (Y.Z.); yangkailin199908@163.com (K.Y.); xdchen@implad.ac.cn (X.C.); pgxiao@implad.ac.cn (P.X.); 2 Key Laboratory of Bioactive Substances and Resources Utilisation of Chinese Herbal Medicine, Ministry of Education, Beijing 100193, China; 3 School of Medical Laboratory, Weifang Medical University, Weifang 261053, China; jieshen0817@163.com

**Keywords:** Huangqin tea, gut microbiota, metabolic transformation, the total flavonoids, interaction

## Abstract

Huangqin tea (HQT), a Non-*Camellia* Tea derived from the aerial parts of *Scutellaria baicalensis*, is widely used in the north of China. The intervention effects of HQT on intestinal inflammation and tumors have been found recently, but the active ingredient and mechanism of action remain unclear. This study aimed to investigate the interactions between the potential flavonoid active components and gut microbiota through culture experiments in vitro combined with HPLC-UV, UPLC-QTOF-MS, and 16S rDNA sequencing technology. The results showed that the HQT total flavonoids were mainly composed of isocarthamidin-7-O-*β*-D-glucuronide, carthamidin-7-O-*β*-D-glucuronide, scutellarin, and others, which interact closely with gut microbiota. After 48 h, the primary flavonoid glycosides transformed into corresponding aglycones with varying degrees of deglycosylation. The composition of the intestinal microbiota was changed significantly. The beneficial bacteria, such as *Enterococcus* and *Parabacteroides,* were promoted, while the harmful bacteria, such as *Shigella,* were inhibited. The functional prediction results have indicated notable regulatory effects exerted by total flavonoids and scutellarin on various pathways, including purine metabolism and aminoacyl-tRNA biosynthesis, among others, to play a role in the intervention of inflammation and tumor-related diseases. These findings provided valuable insights for further in-depth research and investigation of the active ingredients, metabolic processes, and mechanisms of HQT.

## 1. Introduction

The gut microbiota is a complex ecosystem consisting of various microflora that reside in the human gut, which plays a crucial role in digesting food, synthesizing essential vitamins, and regulating the function of the immune system [1]. The composition of the intestinal flora can be influenced by various factors, and diet is widely recognized as the most significant driver in shaping the gut microbiota. In the long term, dietary adjustments were the most effective and healthy approach to modulating and intervening in the intestinal flora [2]. Most Chinese herbal medicines are commonly administered orally, particularly those that can be consumed as tea. The interaction between drugs and the gut microbiota is of significant importance in the processes of digestion, absorption, and metabolism [3,4]. Recently, there has been attention given to the interaction between medicinal substances in traditional Chinese medicine (TCM) and the gut microbiota. However, the specific mechanism by which different types of TCM teas interact with intestinal microorganisms has yet to be revealed [5].

Huangqin tea (HQT) is primarily derived from the stem and leaves of *Scutellaria baicalensis.* As a common Non-*Camellia* Tea, it has been traditionally used in northern and southwest China and possesses distinct properties. The aboveground parts of *S. baicalensis* have heat-clearing, dampness-reducing, fire-reducing, and detoxifying effects. Research indicates that the aerial parts of *S. baicalensis* mainly contain flavonoids and volatile components [6]. Modern studies demonstrated it had various bioactive properties, such as anti-inflammatory, anti-tumor, analgesic, and antipyretic, as well as anti-resistant pathogenic microorganisms [7,8,9,10,11]. Previous studies have reported that baicalin, a compound found in *S. baicalensis*, can be metabolized to baicalein through the intestinal microflora to exert the chemical prevention of colorectal cancer in vivo [12]. Our group’s previous study found that HQT had an obvious intervention effect in the colorectal precancerous lesions induced by azoxymethane (AMO) in rats. It can modulate the intestinal microflora structure in diseased rats and improve the host’s metabolic disturbance. Therefore, it can be seen that the efficacy of HQT is closely related to the gut microbes, and the primary active constituents of HQT are likely to be flavonoids [13]. However, the specific interaction and underlying mechanism of HQT with intestinal microbes have yet to be fully elucidated. Further research is required to investigate and understand the intricate relationship between HQT and the intestinal microbiota. Clarifying this interaction and mechanism would provide valuable insights into the therapeutic effects of HQT and its potential applications in promoting gut health.

In this study, high-performance liquid chromatography (HPLC), ultra-performance liquid chromatography–quadrupole time-of-flight–mass spectrometry (UPLC-Q-TOF-MS), and 16S rDNA sequencing technology were used to investigate the pharmacodynamic ingredients of HQT and the interaction between normal mouse gut microbes and the total flavonoids. From the perspective of prevention, this study aims to elucidate the material basis of HQT and its significant potential value to prevent colorectal cancer and provide a scientific foundation for the development of functional food based on total flavonoids of HQT.

## 2. Materials and Methods

### 2.1. Plant Materials, Chemicals, and Reagents

The *S. baicalensis* stem and leaf were collected from the Institute of Medicinal Plants, Chinese Academy of Medical Sciences in September 2021. The voucher specimens were identified and verified by Professor Chunnian He from the Institute of Medicinal Plant Development (IMPLAD) and then deposited in Pharmacophylogeny Centre of IMPALD in Beijing, China. Scutellarin, apigetrin, baicalin, and isoscutellarein 8-O-*β*-D-glucuronide were purchased from National Institutes for Food and Drug Control. Isocarthamidin-7-O-*β*-D-glucuronide and carthamidin-7-O-*β*-D-glucuronide were isolated from *S. baicalensis* in our laboratory and achieved a purity >95% after UV, IR, and NMR detection [6]. For UPLC-Q-TOF-MS and HPLC-UV analysis, chromatographic-grade methanol, acetonitrile, and formic acid were purchased from Thermo Fisher Scientific (Waltham, MA, USA). All solutions were prepared with ultrapure water (Milli-Q, Millipore Company, Billerica, MA, USA). AB—8 macroporous resin was purchased from Shanghai Macklin Biochemical Co., Ltd. (Shanghai, China). GAM anaerobic culture medium, anaerobic culture bag, anaerobic gas production package, and anaerobic indicator were purchased from Qingdao Hi-tech Industrial Park Hope Bio-technology Co., Ltd. (Qingdao, China). A total of 10 male Kunming mice (8 weeks) were purchased from Sibeifu Biotechnology Co., Ltd. (Beijing, China). The experimental animals were provided free access to food and water at a constant temperature (25 ± 2 °C) and humidity (55 ± 10%) on a 12 h light/dark cycle for 3 weeks. 

### 2.2. The HQT Total Flavonoids Preparation

The stems and leaves of *S. baicalensis* were cut into 1–2 cm and dried. Plant samples (1 kg) were soaked in 1200 mL of deionized water for 30 min, heated and refluxed for 60 min, and filtered. Then, they were extracted twice with 1000 mL and 800 mL of deionized water, combined with filtrate, vacuum-concentrated to 0.42 g∙mL^−1^ (SP Genevac Rocket Synergy, Genevac, UK), and concentrated hydrochloric acid was added to adjust the pH to 2–3. The AB-8 macroporous resin was used to purify the solution after loading the liquid. After adding, the liquid absorbed for 2 h. Sugars and other impurities were removed using deionized water (9 L), followed by elution with 70% ethanol (15 L). The eluent was collected, and the ethanol was recovered under reduced pressure. The samples were frozen in −20 °C refrigerator overnight for 24 h, then freeze-drying (Lyovapor L-300, BUCHI, Flawil, Switzerland) to obtain the HQT total flavonoids (125.0 g, yield 12.50%).

### 2.3. Determination Content of HQT Total Flavonoids

The major chemical components of HQT total flavonoids were analyzed using HPLC (Thermo U3000, Waltham, MA, USA) [6], as well as the content of six major flavonoids—isocarthamidin-7-O-*β*-D-glucuronide, carthamidin-7-O-*β*-D-glucuronide, scutellarin, apigetrin, baicalin, and isoscutellarein 8-O-*β*-D-glucuronide—in the HQT total flavonoids.

### 2.4. The Metabolic Transformation of Gut Microbiota In Vitro

The total flavonoids solution, 2.5 mg/mL, was prepared using sterile water. Scutellarin was prepared using DMSO and subsequently diluted to 0.25 mg/mL with sterile water. To ensure sterility, the GAM anaerobic culture medium and other experimental materials underwent sterilization in autoclave sterilizer (MLS 375, Panasonic, Osaka, Japan) at 121 °C and 0.120 MPa for 15 min. The fresh feces from healthy mice were collected and mixed with sterile saline at 1:4 (*w*/*v*) and centrifuged at 4000 r/min (TG 16 G, Tianjin, China) for 10 min (4 °C). After centrifugation, the supernatant was removed, mixed with culture medium (1:9, *v*/*v*), and incubated in an incubator (SPX-50B, Shanghai Lichen Technology Co., Ltd., Shanghai, China) for 48 h (37 °C) to obtain the blank incubation solution. Additionally, the blank culture solution was prepared using normal saline according to the same method described above. The HQT total flavonoids solution was mixed with the incubation solution (1:8, *v*/*v*), then further cultured as the total flavonoids conversion group (Group 1). Other groups were prepared in the same proportion. The scutellarin control group (Group 2) combined the scutellarin control solution with the gut microbiota incubation solution. The blank control group (Group 3) consisted of sterile water mixed with the intestinal incubation solution. The sterile total flavonoids control group (Group 4) was created by combining the total flavonoids solution with the blank medium. The blank sterile group (Group 5) was formed by mixing sterile water with the blank medium. Lastly, the drug group (Group 6) was prepared by combining the total flavonoids solution with sterile water. For each group, three samples were prepared in parallel. The groups were placed in anaerobic bags and incubated at 37 °C. Samples were collected at various time points: 0 h, 4 h, 24 h, 36 h, 48 h, and 72 h. For each time point, 750 μL gut microbiota samples were taken and mixed with an equal volume of ice methanol. The mixture was centrifuged at 13,000 r/min for 10 min (4 °C), and the resulting supernatant was collected for analysis using HPLC under the same chromatographic conditions described in Section 2.3. Additionally, groups 1, 2, and 3 were also analyzed using UPLC-Q-TOF-MS [14]. After sampling, the samples were separated and stored at −80 °C.

### 2.5. 16S rDNA Sequencing Technology

16S rRNA amplicon sequencing was performed for three parallel 0 h and 48 h samples of the total flavonoids conversion group (Group 1), the scutellarin control group (Group 2), and the blank control group (Group 3) under item “2.4”. Total DNA was extracted from the samples, and the full length of 16S rDNA was amplified using specific primers. After purifying and quantifying the amplified products, the full-length sequences were selected for sequencing using the universal primer 27F(AG RGTTTGA TYNTGGCTCAG and 1492R (TASGGHTACCTTGTTASGACTT) [15]. The PCR amplification was performed using the New England Biolabs Phusion^®^ High-Fidelity PCR Master Mix with GC Buffer and the efficient fidelity enzyme. After amplification, library construction was carried out. The library was then subjected to quality detection using Qubit, and the insert size was assessed using the Agilent 2100 system, followed by sequencing using the PacBio platform. After sequencing the data using the PacBio platform, the DADA2 method was employed for data processing. This involved removing, correcting, reducing noise, and eliminating chimeras to obtain the ASV (amplicon sequence variant) sequences and their corresponding abundance information. To ensure the accuracy and reliability of the information analysis results, the original data underwent assembly and filtering to obtain valid data.

### 2.6. Statistical Analysis

The data were presented as the mean ± standard deviation (SD). Statistical analysis was conducted using IBM SPSS 23 software (IBM Software, New York, NY, USA), and the graphs were generated using GraphPad Prism 9 software (GraphPad Software, San Diego, CA, USA). The statistical significance of the data was assessed using one-way ANOVA followed by Duncan’s test. A value of *p* < 0.05 was considered statistically significant.

## 3. Results

### 3.1. The Content Determination of the Total Flavonoids Sites of HQT

**Figure 1:**
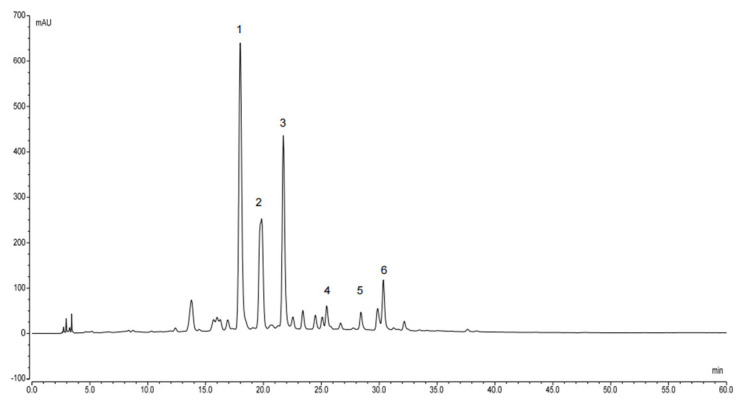
The HPLC chromatogram of of six major flavonoids in the total flavonoid sites of HQT. (1: isocarthamidin-7-O-*β*-D-glucuronide, 2: carthamidin-7-O-*β*-D-glucuronide, 3: scutellarin, 4: apigetrin, 5: baicalin, 6: isoscutellarein 8-O-*β*-D-glucuronide).

The contents of six major flavonoids in the total flavonoids sites of HQT were determined by HPLC. Table 1 and Figure 1 showed the content determination results of isocarthamidin-7-O-*β*-D-glucuronide, carthamidin-7-O-*β*-D-glucuronide, scutellarin, apigetrin, baicalin, and isoscutellarein 8-O-*β*-D-glucuronide. As can be seen in Table 1, the content of total flavonoids in HQT exceeded 54.51%.

### 3.2. The Metabolic Transformation of the Mouse Gut Microbiota In Vitro

**Figure 2:**
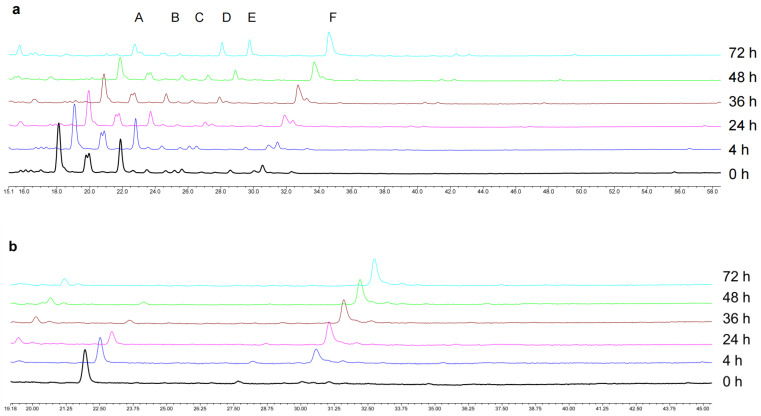
The HPLC plot of the total flavonoids conversion group and the scutellarin control group at different time points. (**a**) The HPLC plot of the total flavonoids conversion group; (**b**) The HPLC plot of the scutellarin control group. (Peak A: isocarthamidin-7-O-*β*-D-glucuronide. Peak B: carthamidin-7-O-*β*-D-glucuronide. Peak C: scutellarin. Peak D, E and F: These were the newly generated peaks during the transformation process).

In this study, the anaerobic temperature incubation system of mouse gut microbiota was established successfully in vitro. The results presented in Table 2 demonstrate a decrease in the concentration of total flavonoids and scutellarin in the incubation solution after 72 h in the total flavonoids conversion group (Group 1) and the scutellarin control group (Group 2). However, the concentration of total flavonoids in the blank culture medium remained relatively stable during the same period. Additionally, it can be seen that the peaks of isocarthamidin-7-O-*β*-D-glucuronide, carthamidin-7-O-*β*-D-glucuronide, and scutellarin were significantly decreased with time in Figure 2a. This decrease was accompanied by the gradual emergence of peaks D, E, and F, which exhibited a substantial increase in content. Furthermore, Figure 2b shows that the content of scutellarin in Group 2 gradually decreased, and the new peak gradually increased.

**Figure 3:**
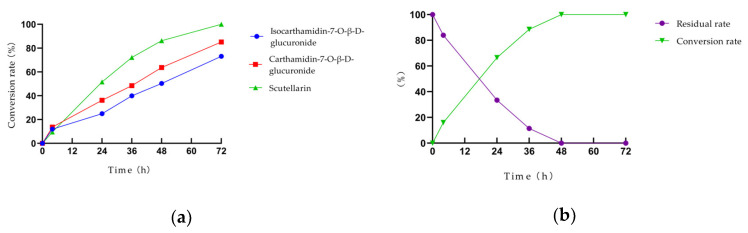
The conversion rate curve of drugs in the gut microbiota incubation solution. (**a**) The conversion rate curve of the three major flavonoid components of the total flavonoids conversion group; (**b**) The conversion rate curve of scutellarin in the scutellarin control group.

According to the determination results presented in Section 3.1, isocarthamidin-7-O-*β*-D-glucuronide, carthamidin-7-O-*β*-D-glucuronide, and scutellarin accounted for 91.1% of the total flavonoids of HQT. Therefore, to better monitor the changes in the total flavonoids content, these three compounds were selected as the key components to calculate the conversion rate of total flavonoids in the gut microbiota incubation solution and draw the time–conversion rate curve in the study. Figure 3a illustrates that the conversion rate curve of these three flavonoid components exhibited an increasing trend. Notably, the conversion rate of scutellarin was higher than that of the other two components in the total flavonoids, reaching over 80% after 48 h of incubation, while the conversion rate of isocarthamidin-7-O-*β*-D-glucuronide was the lowest. In Figure 3b, the initial content of scutellarin at 0 h was set as 100%; almost all of the scutellarin had been converted into its microbiota metabolites after 48 h.

### 3.3. Metabolite Identification of Intestinal Microbiota Based on UPLC-Q-TOF-MS

**Figure 4:**
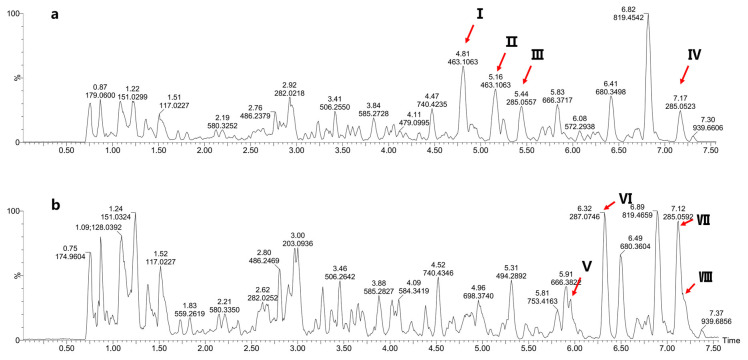
The BPI plots of the total flavonoids conversion group (Group 1) at 0 h and 72 h. (**a**) The BPI plot of Group 1 at 0 h; (**b**) The BPI plot of Group 1 at 72 h. (The numbers of peaks in figure is the same as in Table 3).

To investigate the metabolites of total flavonoids in the gut microbiota, we conducted UPLC-Q-TOF-MS analysis and generated a base peak ion (BPI) plot of the sample, which is presented in Figure 4. Figure S1 shows the BPI plots of the blank control group. Table 3 provides detailed information on the formula, retention time, molecular weight, and secondary fragmentation of the compounds. Upon identification and analysis, the main flavonoids were transformed into their respective components. Figure 4a demonstrates that during the initial stage, four components were detectable: isocarthamidin-7-O-*β*-D-glucuronide, carthamidin-7-O-*β*-D-glucuronide scutellarin, and isoscutellarein 8-O-*β*-D-glucuronide. Their numbers were given as I, Ⅱ, Ⅲ, and Ⅳ, respectively. Most flavonoid components are commonly found in the form of glycosides. Previous studies have demonstrated that deglycosylation of glucoside flavonoids mainly occurs in the gut [16]. Figure 4b and Table 3 shows that four flavonoid components lose one part of glucuronide, transformed into its corresponding glycoside, isocarthamidin, carthamidin, scutellarein, and isoscutellarein.

### 3.4. The Analysis of Gut Microbiotaoperational Taxonomic Units (OTUs)

**Figure 5:**
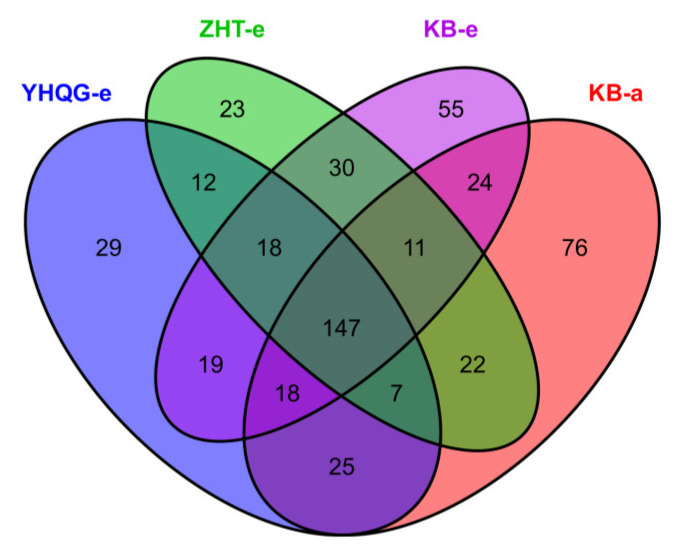
The OTUs Venn diagram for each group. (KB-a: the blank control group at 0 h, KB-e: the blank control group at 48 h, ZHT-e: the total flavonoids conversion group at 48 h, YHQG-e: the scutellarin control group at 48 h).

To analyze the effect of the total flavonoids and scutellarin on the intestinal microbiota of mice, the characteristics and common taxa of the different treatment groups are represented in Venn diagrams, as shown in Figure 5. After 48 h, the OTUs decreased from 330 to 322 in the blank control group. This suggested that a few intestinal microbiota may be inhibited upon separation from the intestinal environment. The OTUs in both the total flavonoids conversion group and the scutellarin control group decreased further after incubation, with an 18% decrease in the former and a 17% decrease in the latter. This observation suggested that total flavonoids and scutellarin may exert antibacterial effects on certain intestinal microbiota in vitro.

### 3.5. The Intestinal Microbiota α Diversity and β Diversity Analysis

**Figure 6:**
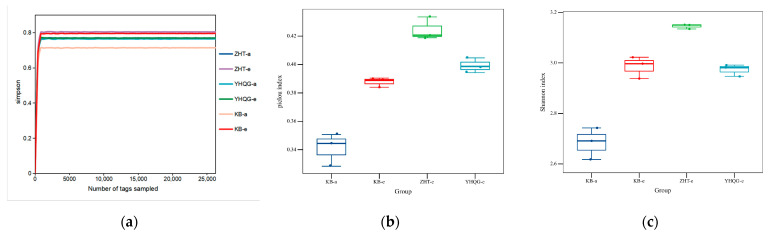
The gut microbial α diversity analysis. (**a**) The dilution curve of Simpson index; (**b**) The box plots of Pielou index in different groups; (**c**) The box plots of Shannon index in different groups. (KB-a: the blank control group at 0 h, KB-e: the blank control group at 48 h, ZHT-a: the total flavonoids conversion group at 0 h, ZHT-e: the total flavonoids conversion group at 48 h, YHQG-a: the scutellarin control group at 0 h, and YHQG-e: the scutellarin control group at 48 h).

Alpha diversity analysis was conducted to assess the sequencing volume’s adequacy in capturing all taxa and indirectly measuring species richness in the sample. In Figure 6a, the dilution curve’s slope approached 0, indicating that the sequencing depth effectively accounted for all species in the sample. Subsequently, the α diversity index was analyzed by using the Tukey test and the Kruskal–Wallis rank-sum test. The results are shown in Figure 6b,c as well as Table S1.

The pielou and Shannon indexes of the three groups were increased after 48 h of culture. Specifically, the indexes in ZHT-e presented significance (*p* < 0.05). These findings suggested that the total flavonoids in HQT may have the potential to enhance the uniformity of species distribution and the overall community diversity of the intestinal microbiota in normal mice.

**Figure 7:**
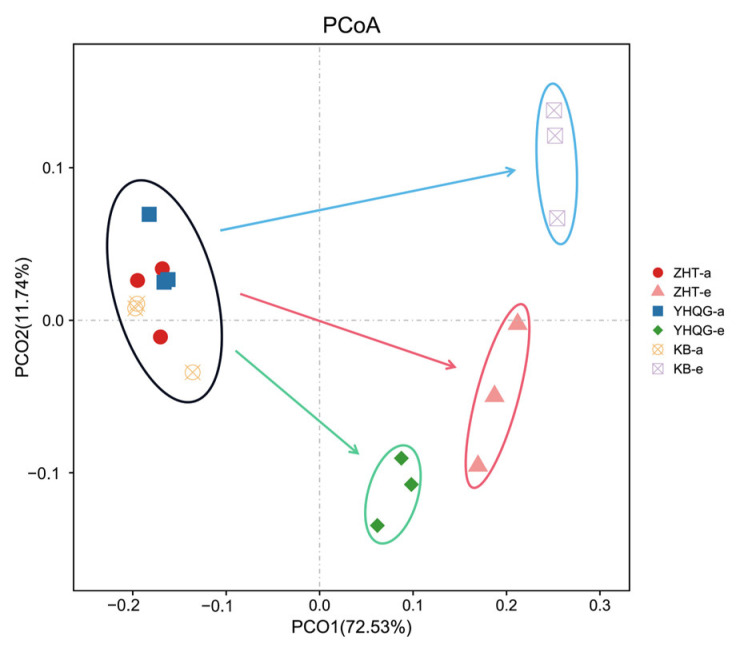
The PCoA analysis of the gut microbiota based on the species abundance (*n* = 3). (KB-a: the blank control group at 0 h, KB-e: the blank control group at 48 h, ZHT-a: the total flavonoids conversion group at 0 h, ZHT-e: the total flavonoids conversion group at 48 h, YHQG-e: the scutellarin control group at 0 h, and YHQG-e: the scutellarin control group at 48 h).

Beta diversity analysis can assess the variation in microbial composition among different samples. In this study, we analyzed the composition of mouse intestinal microbiota using principal coordinate analysis (PCoA). As shown in Figure 7, each group formed clusters at 0 h, suggesting that the initial structural composition of each group was relatively similar during the early stage of transformation. After 48 h of anaerobic culture, the blank control group, the total flavonoids conversion group, and the scutellarin control group exhibited distinct clusters in comparison to the three groups at 0 h. This suggested that the bacterial composition of each group underwent changes during the culture period, and there were significant differences between the groups. This finding further supported that the composition of gut microbes underwent significant changes following treatment with total flavonoids and scutellarin compared to the blank control group.

### 3.6. Analysis of the Microbiota Community Composition

The analysis of the species distribution of mouse gut microbiota showed that the distribution of the top 10 species in relative abundance at the phylum level and genus level varied after 48 h of incubation. Hence, the discrepancies in the gut microbiota among the various groups were visualized.

**Figure 8:**
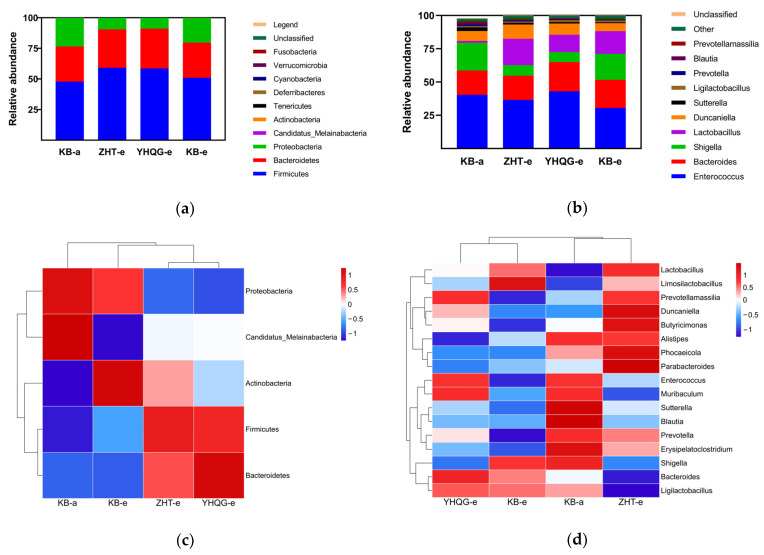
The analysis of relative abundance of gut microbiota species in three groups at phylum and genus levels. (**a**) The species stacking map of the relative abundance at the phylum level. (**b**) The species stacking map of the relative abundance at the genus level. (**c**) The heatmap of differential gut bacterial taxa at phylum level. (**d**) The heatmap of differential gut bacterial taxa at genus level. (KB-a: the blank control group at 0 h, KB-e: the blank control group at 48 h, ZHT-e: the total flavonoids conversion group at 48 h, and YHQG-e: the scutellarin control group at 48 h).

In Figure 8a, at the taxonomic phylum level, *Firmicutes* and *Proteobacteria* in the total flavonoids conversion group and scutellarin control group changed significantly compared to the blank control group. In addition, at the taxonomic genus level, *Enterococcus* was the most abundant genus in each group in Figure 8b. *Enterococcus* in the blank control group and the total flavonoids conversion group significantly reduced after 48 h (*p* < 0.01) compared with the blank control group at 0 h in Figure 8d. Interestingly, in the scutellarin control group, the abundance of *Enterococcus* remained relatively stable throughout the period. In Figure 8d, after the culture, the levels of *Enterococcus* were found to be highest in the YHQG-e group, followed by the ZHT-e group, and finally, the KB-e group. These findings suggested that scutellarin can help to keep the abundance of *Enterococcus* within the gut microbiota. Apart from its probiotic potential, studies have shown that *Enterococcus* can synthesize exopolysaccharides with various bioactive properties, which possess antioxidant, antibacterial, anti-biofilm, antitumor, immunological, and anti-diabetic activities [17]. This indicated that *Enterococcus* has a wide range of potential health benefits and therapeutic applications. Notably, the levels of *Shigella* were significantly reduced in the total flavonoids conversion group and scutellarin control group. The finding suggested that total flavonoids and scutellarin have inhibitory effects on *Shigella*. According to Figure 8d, it is also found that the abundance of *Parabacteroides*, *Butyricimonas*, and *Phocaeicola* increased following intervention with total flavonoids compared to the other groups. Additionally, the intervention of scutellarin led to an increase in the abundance of *Bacteroides*, *Ligilactobacillus*, *Enterococcus*, and *Muribaculum*.

### 3.7. The Differential Bacterial Screening Results

The linear discriminant analysis (LEfSe, linear discriminant analysis effect size) combining the rank sum test and discriminant analysis was used to identify significantly changing microbial taxa. The cladogram illustrated the taxonomic distribution of labeled species in each group, with larger circles indicating a higher degree of enrichment. The threshold for linear discriminant analysis (LDA) was set at a value greater than 4, with *p* < 0.05.

**Figure 9:**
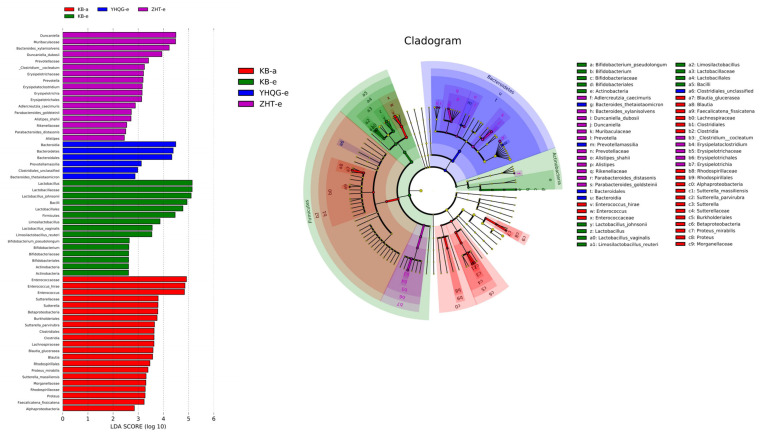
The LEfSe analysis bar chart (**left**) and evolutionary clade chart (**right**) (LDA > 2 and *p* < 0.05). In the evolutionary clade gram, circles radiating from inside to outside represent the taxonomic level from phylum to genus (or species). Each small circle at different taxonomic level represents a classification at the level; the small circle diameter is proportional to the relative abundance size. Red nodes represent differential species biomarkers that played an important role in the red group (KB-a: the blank control group at 0 h), green nodes represent differential species biomarkers that played an important role in the green group (KB-e: the blank control group at 48 h), blue nodes represent differential species biomarkers that played an important role in the blue group (YHQG-e: the scutellarin control group at 48 h), and purple nodes represent differential species biomarkers that played an important role in the purple group (ZHT-e: the total flavonoids conversion group at 48 h).

In this study, 59 distinct bacterial taxa were selected, which encompassed 3 phyla, 7 classes, 7 orders, 12 families, 12 genera, and 18 species (refer to Figure 9). The length of each bar in the chart indicates the effect size of the corresponding taxa. Prior to the transformation, *Enterococcaceae*, *Sutterellaceae*, *Lachnospiraceae*, and *Morganellaceae* were predominantly enriched. After 48 h, the blank control group exhibited the highest abundance of *Lactobacillaceae* and *Bifidobacteriaceae*. Meanwhile, the total flavonoids conversion group was predominantly enriched with *Muribaculaceae*, *Bacteroidaceae*, *Rikenellaceae*, *Prevotellaceae,* and *Erysipelotrichaceae*. The scutellarin control group primarily influenced *Prevotellamassilia.*

### 3.8. The Prediction Results of Gut Microbial Function

Tax4Fun [18] was a functional prediction tool based on the SILVA [19] database. It primarily focuses on the functional annotation of sequenced and annotated prokaryotic genomes in the KEGG database for functional prediction for the OTUs information. The SILVA database, which is rapidly updated, offers a significant advantage in terms of the accuracy of functional prediction results. Consequently, it enables us to gain more comprehensive insights into the gut microbiota community.

In KEGG pathway analysis, the functions of lever 1 mainly included metabolism, cellular processes, and human diseases. Level 2 involved membrane transport, carbohydrate metabolism, and amino acid metabolism. Level 3 were transporters, ABC transporters, and ribosomes. In Figure S2, we identified six primary differential pathways according to the KEGG database. Additionally, we found 36 secondary differential pathways and 259 tertiary differential pathways within the same database. According to Figure S2, compared with the blank control group, the scutellarin control group exhibited significant up-regulation in several metabolic pathways, including Purine metabolism, Aminoacyl-tRNA biosynthesis, Pyrimidine metabolism, two-component system, starch and sucrose metabolism, and ribosome. In the total flavonoids conversion group, several metabolic pathways, including Aminoacyl-tRNA biosynthesis, Nicotinate and nicotinamide metabolism, homologous recombination, Histidine metabolism, and DNA replication, exhibited significant differences compared with the blank control group.

## 4. Discussion

HQT derived from the aerial parts part of *Scutellaria baicalensis*, which had significant application potential. It has a longstanding tradition of being consumed as a tea in the northern and southwestern regions of China. Our previous experiments found that HQT had an obvious intervention effect in the colorectal precancerous lesions induced by AMO in rats. Additionally, the inhibitory effect on aberrant crypt foci (ACF) formation was primarily attributed to the influence of HQT on the intestinal microflora [13]. Therefore, we explored the interaction of total flavonoids and normal mouse intestinal microflora based on the in vitro transformation system in this study.

The predominant components of the total flavonoids in HQT were isocarthamidin-7-O-*β*-D-glucuronide, carthamidin-7-O-*β*-D-glucuronide, and scutellarin. We investigated the transformation of total flavonoids by the intestinal microflora of normal mice through anaerobic culture in vitro. According to the conversion curve, the conversion rate of scutellarin consistently exceeded that of isocarthamidin-7-O-*β*-D-glucuronide and carthamidin-7-O-*β*-D-glucuronide in the total flavonoids conversion group. Furthermore, after 72 h, scutellarin in the total flavonoids was nearly completely transformed into its metabolites. Further, the UPLC-Q-TOF-MS analysis of the total flavonoids conversion group revealed a conversion of the primary flavonoid glycosides in the total flavonoids into their respective glycosides within the bacterial culture system. Current findings indicated that the metabolic processes conducted by intestinal microflora typically involve deglycosylation, demethylation, reduction, and cyclic fission reactions [20,21]. The biotransformation of flavonoids in vivo was a highly complex process that encompassed the involvement of multiple organs and diverse enzymes. According to recent investigations, upon entering and being transported into the liver, the drug underwent further coupling reactions, such as sulfation and methylation, resulting in the formation of many conjugates [16]. Most *O*-glycosides of flavonoids underwent *O*-deglycosylation when acted upon by intestinal bacteria [22]. In this study, isocarthamidin-7-O-*β*-D-glucuronide, carthamidin-7-O-*β*-D-glucuronide, scutellarin, and isoscutellarein 8-O-*β*-D-glucuronide detected in 0 h group by UPLC-Q-TOF-MS were *O*-glycosides. Through fragment ion identification, it was found that in the intestinal bacteria culture system in this study, the *O*-glycoside compounds in HQT were converted into corresponding glycoelements by deglycosylation, and this result was consistent with the previous report. Most natural flavonoids predominantly existed as glycosides, rendering them poorly absorbed in the small intestine. Consequently, they transited to the colon in their glycoside form and hydrolyzed into aglycones by the intestinal microflora. Subsequently, these compounds underwent further metabolism, leading to the formation of various metabolites and phenolic acids, which could be more efficiently absorbed by the human body [23]. Hence, the total flavonoids of HQT facilitated their absorption by the gut microbiota, obtaining better effects. This observation underscored the pivotal role played by the intestinal flora in this process.

Based on the sequencing results, the total flavonoids and scutellarin led to slightly lower OUTs compared to the blank control group. This observation suggested that total flavonoids and scutellarin may exert a certain inhibitory effect on individual bacteria [24]. In the warm incubation system outside the body, the absence of regulatory mechanisms for intestinal environment homeostasis led to slight inhibitory effects at the OUTs level. The Pielou and Simpson indices of the total flavonoids conversion group revealed higher values compared to those of the blank control group in Figure 6b,c after 48 h, suggesting that the distribution of the mouse intestinal microbiota may be more uniform in that group. According to the PCoA, the intestinal microbiota structure exhibited significant changes following the intervention of total flavonoids and scutellarin. These findings suggested that the intervention of total flavonoids can facilitate a more uniform distribution of microflora and regulate the microflora structure. To gain deeper insights into the structure of the microflora, we conducted an analysis of the microbial community composition.

At the phylum level, *Firmicutes* and *Proteobacteria* exhibited similar variation tendencies in the total flavonoids conversion group and scutellarin control group. This observation suggested that the effect of total flavonoids and scutellarin on the normal gut microbiota was moderate. This finding further reflected that consuming HQT did not excessively disrupt the balance of normal intestinal microbiota in the human body. At the genus level, total flavonoids and scutellarin increased the abundance of *Enterococcus* while inhibiting the growth of *Shigella*. *Enterococcus* is a naturally occurring component of the human microbiota and is abundant in the intestine, where it plays a crucial role in the digestive system [25]. In addition, *Enterococcus* species were commonly utilized as probiotic food additives or recommended as supplements for the management of intestinal dysbiosis and other ailments [26]. Conversely, extensive research indicated that *Shigella* was a prevalent pathogen responsible for bacterial dysentery in humans. It had the ability to evade immune defenses quickly and induce hemorrhagic diarrhea and severe intestinal inflammation [27]. The total flavonoids and scutellarin might promote the growth of beneficial bacteria, maintain the stability of the intestinal environment, have a certain inhibitory effect on pathogenic bacteria, and reduce the occurrence of digestive system diseases.

In Figure 8d, the abundance of *Parabacteroides*, *Butyricimonas*, and *Phocaeicola* was upregulated under the intervention of total flavonoids. *Parabacteroides* are key members of the human gut microbiota, which have the physiological characteristics of carbohydrate metabolism and secretion of short-chain fatty acids (SCFAs). As a potential probiotic bacterium, its species has potential therapeutic effects in metabolic, inflammatory, and neoplastic diseases [28,29,30,31]. The upregulation of *Butyricimonas* abundance can enhance the function of fatty acid biosynthesis, increase the production of SCFAs, and promote the synthesis of butyrate [32]. Recent studies have found that *Phocaeicola* strains can help maintain the epithelial barrier by regulating cytokine levels and the secretion of SCFAs, thereby improving dextran sodium sulfate-induced colitis in mice [33]. And studies have found that increasing the production of SCFAs contributes to the protective effects of probiotics against intestinal and brain health [34]. The results of this study showed that the total flavonoids may effectively increase the abundance of dominant bacteria, enable the intestine to better secrete SCFAs, enhance the protective effect on the intestine, maintain the integrity of the intestinal epithelial barrier, and promote the synthesis of butyrate and provide energy for the intestine. In the previous experiments of our lab, we found that the abundance of related bacteria producing both butyrate and SCFAs increased under the intervention of HQT, which was similar to the results in this study [14]. After transformation, the abundance of *Bacteroides*, *Ligilactobacillus*, *Enterococcus*, and Muribaculum was upregulated in the scutellarin control group. Studies reported that *Bacteroides* played a key role in regulating the human immune system, mainly in maintaining the stability of the immune system, anti-inflammatory and anti-tumor systems, and other aspects [35]. As beneficial bacteria, many *Ligilactobacillus* strains exhibited functional properties with health benefits, such as antimicrobial activity, immune effects, and the ability to modulate the gut microbiota [36]. Scutellarin may exert an anti-inflammatory function by promoting the abundance of beneficial bacteria and regulating the immune system function, as well as playing a certain preventive or therapeutic role in the premalignant stage.

The LEfSe analysis showed that *Muribaculaceae*, *Bacteroidaceae*, *Rikenellaceae*, and *Prevotellaceae* were the dominant bacteria in the total flavonoids conversion group, most of which were beneficial bacteria. It has been reported that *Muribaculaceae* can exert beneficial effects on intestinal ecology by regulating immunity and intestinal homeostasis, increasing the output of intestinal beneficial metabolites, prolonging the life span of mice, and inhibiting CD8 + T cell activation to tolerate immune stimulation, and is negatively correlated with inflammatory response [37,38]. In addition, experiments have proved that *Rikenellaceae* and *Bacteroidaceae*, as probiotics, had good anti-inflammatory effects, and *Rikenellaceae* can also protect cells from oxidative stress by neutralizing cytotoxic reactive oxygen species and enhancing the barrier function of intestinal epithelial cells [39,40,41]. *Erysipelotrichaceae* was important in the immune response, which was highly immunogenic. The bacteria can also produce SCFAs, such as butyric acid, through dietary fiber fermentation [42]. SCFAs induced protective immune responses by producing anti-inflammatory cytokines TGF- *β* and IL-10 and enhancing epithelial barrier function [43,44]. The scutellarin control group mainly included the dominant gut microbiota, such as *Prevotellaceae* and *Clostridiales*. Animal experiments have demonstrated that *Prevotellaceae* can inhibit the development of inflammatory arthritis in mice and regulate the host immune response [45]. *Clostridiales*, on the other hand, are beneficial for overall health and exhibit an effective anti-tumor response independent of anti-PD-1 immunotherapy by activating CD8 + T cells [46,47]. Therefore, scutellarin may potentially enhance its anti-inflammatory function by promoting the growth of these dominant bacteria, thereby aiding in the regulation and activation of the immune system.

The gut microbial function prediction results showed that the YHQG group was significantly up-regulated in several metabolic pathways, including Purine metabolism, Aminoacyl-tRNA biosynthesis, Pyrimidine metabolism, and starch and sucrose metabolism. It has been demonstrated that Lactobacillus plays a key role in the Purine metabolism pathway [48]. Based on the analysis of gut microbiota structure, it was observed that the total flavonoids conversion group and scutellarin control group increased *Lactobacillus* abundance after transformation. Hence, we speculated that the main component regulating the Purine metabolism pathway in the total flavonoids group was scutellarin. Relative to the KB group, the total flavonoids conversion group showed significant upregulation in several pathways, including Aminoacyl-tRNA biosynthesis, Nicotinate and nicotinamide metabolism, homologous recombination, Histidine metabolism, and DNA replication. Multiple studies have provided evidence that Aminoacyl-tRNA biosynthesis was a biosynthetic pathway enriched with metabolites associated with CRC [49]. The involvement of relevant synthetases in Aminoacyl-tRNA biosynthesis implies that HQT total flavonoids might disrupt CRC development by influencing these synthetases within the Aminoacyl-tRNA biosynthesis pathway.

## 5. Conclusions

In this study, the total flavonoids were obtained through a process involving water frying and macroporous resin purification. The quantification of the main flavonoids was performed using HPLC (yield 12.50%, content 54.51%). The present study investigated the interaction between the HQT total flavonoids and the gut microbiota. Moreover, the transformation analysis demonstrated that the cultured intestinal bacteria were capable of metabolizing the total flavonoids and converting them into the corresponding aglucons. According to the results of intestinal group sequencing, it was observed that the total flavonoids have the ability to enhance the growth of dominant intestinal microflora. Additionally, they significantly increased the abundance of beneficial bacteria such as *Enterococcus* and *Lactobacillus*. This finding suggested that total flavonoids can potentially contribute to regulating the human immune response, exhibiting antimicrobial and antioxidant properties, as well as demonstrating anti-tumor effects [50].

The functional prediction results revealed significant regulatory effects of total flavonoids and scutellarin on various pathways, including Purine metabolism, Aminoacyl-tRNA biosynthesis, Pyrimidine metabolism, Nicotinate and nicotinamide metabolism, homologous recombination, and Histidine metabolism. These findings provided valuable insights for further in-depth research and investigation. According to this study, it was found that the total flavonoids of HQT could potentially serve as the primary active component responsible for its intervention effect on the intestinal microbiota. This finding not only provides a new perspective to reveal the mechanism behind the prevention of inflammatory and tumor-related diseases, particularly colorectal cancer (CRC), through interventions in the gut microbiota but also offers a fresh perspective on this topic.

## Figures and Tables

**Table 1:** The contents of six major flavonoids in the total flavonoid sites of HQT.

NO.	Compound	Content (mg/g)
1	Isocarthamidin-7-O-*β*-D-glucuronide	242.99 ± 9.16
2	Carthamidin-7-O-*β*-D-glucuronide	161.23 ± 15.75
3	Scutellarin	91.50 ± 3.30
4	Apigetrin	9.79 ± 1.57
5	Baicalin	7.48 ± 0.65
6	Isoscutellarein 8-O-*β*-D-glucuronide	32.08 ± 3.71

**Table 2:** The transformation concentration of total flavonoids and scutellarin in the mouse intestinal microflora incubation solution for 72 h. (Group 1: the total flavonoids conversion group, Group 2: the scutellarin control group, and Group 4: the sterile total flavonoids control group).

Time (h)	Concentrations (μg/mL)		
	Group 1	Group 2	Group 4
0	140.67	14.07	140.67
4	124.08	11.81	139.22
24	91.60	4.71	139.57
36	67.54	1.61	139.88
48	51.69	0	139.95
72	23.47	0	139.51

**Table 3:** The fragmentation of the compounds of the total flavonoids conversion group at 0 h and 72 h.

No.	Compound	Formula	Retention Time (min)	*m*/*z*	[M-H]-	Fragmentation Ions	Intensity 0 h/72 h
I	Isocarthamidin-7-O-*β*-D-glucuronide	C_21_H_20_O_12_	4.810	464.1070	463.1063	287.0677	523,873/2604
II	Carthamidin-7-O-*β*-D-glucuronide	C_21_H_20_O_12_	5.160	464.1070	463.1063	287.0711, 202.1172	513,752/2442
III	Scutellarin	C_21_H_18_O_12_	5.438	462.0935	461.0949	285.0557	182,889/1087
IV	Isoscutellarein-8-*β*-glucuronide	C_21_H_18_O_12_	7.166	462.0935	461.0905	285.0552	266,004/721
V	Isocarthamidin	C_15_H_12_O_6_	5.945	288.0745	287.0746	-	3060/238199
VI	Carthamidin	C_15_H_12_O_6_	6.324	288.0745	287.0746	-	3964/449457
VII	Scutellarein	C_15_H_12_O_6_	7.116	286.0625	285.0591	-	6206/367239
VIII	Isoscutellarein	C_15_H_12_O_6_	7.187	286.0591	285.0591	253.064	18,127/15,2855

## Data Availability

The data used to support the findings of this study can be made available by the corresponding author upon request.

## Supplementary Materials

The following supporting information can be downloaded at: https://www.mdpi.com/article/10.3390/foods12244410/s1, Supplementary Figure S1. The BPI plots of the blank control group (Group 3) at 0 h and 72 h. (a) The BPI plot of Group 3 at 0 h; (b) The BPI plot of Group 3 at 72 h; Supplementary Figure S2. Analysis of TaxFun4 function prediction differences in KEGG pathway (A: After 48 h, variation analysis between KB-e (the blank control group at 48 h) and YHQG-e (the scutellarin control group at 48 h), B: After 48 h, variation analysis between KB-e (the blank control group at 48 h) and ZHT-e (the total flavonoids conversion group at 48 h); Supplementary Table S1. The α diversity index for each group. (KB-a: the blank control group at 0 h, KB-e: the blank control group at 48 h, ZHT-e: the total flavonoids conversion group at 48 h, and YHQG-e: the scutellarin control group at 48 h).
