# Supplementary figures and images for: Huangqin Tea Total Flavonoids–Gut Microbiota Interactions: Based on Metabolome and Microbiome Analysis
Source: Foods. 2023 Dec 7;12(24):4410. doi: 10.3390/foods12244410 (PMC10742805; doi:10.3390/foods12244410)

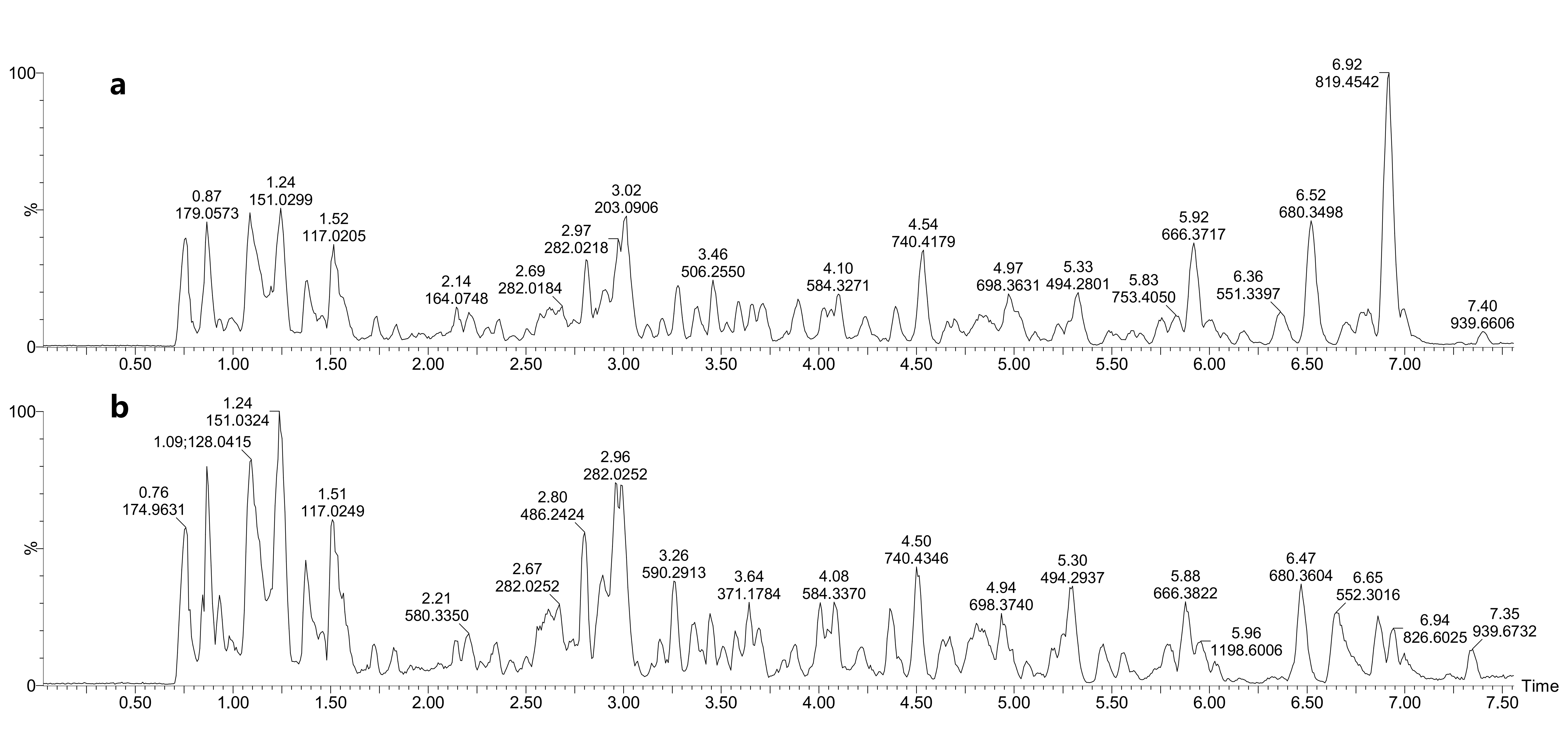

Supplement: Supplementary file 1 [file foods-12-04410-s001.zip › Supplementary Figure S1.png]

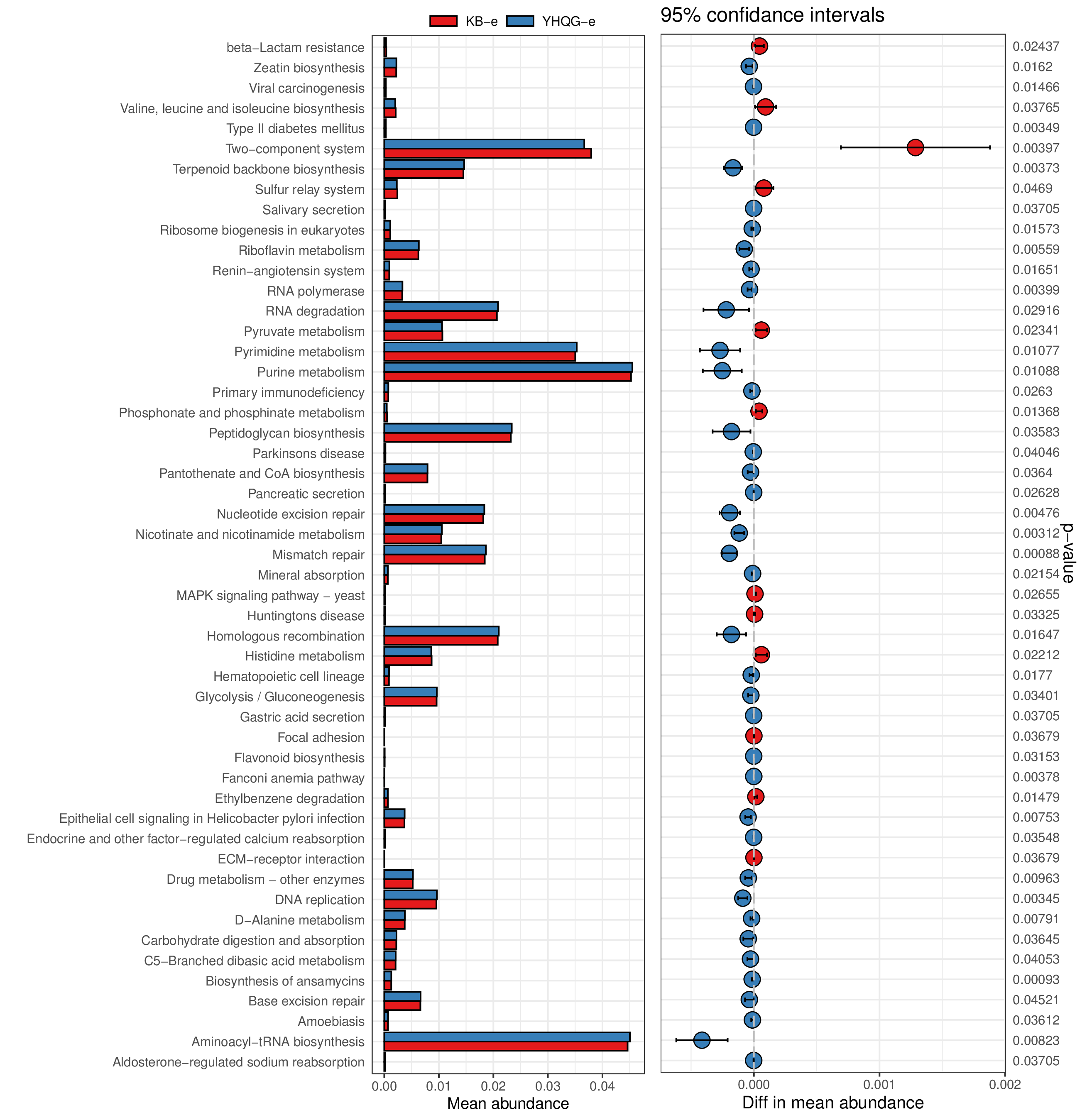

Supplement: Supplementary file 1 [file foods-12-04410-s001.zip › Supplementary Figure S2(a).png]

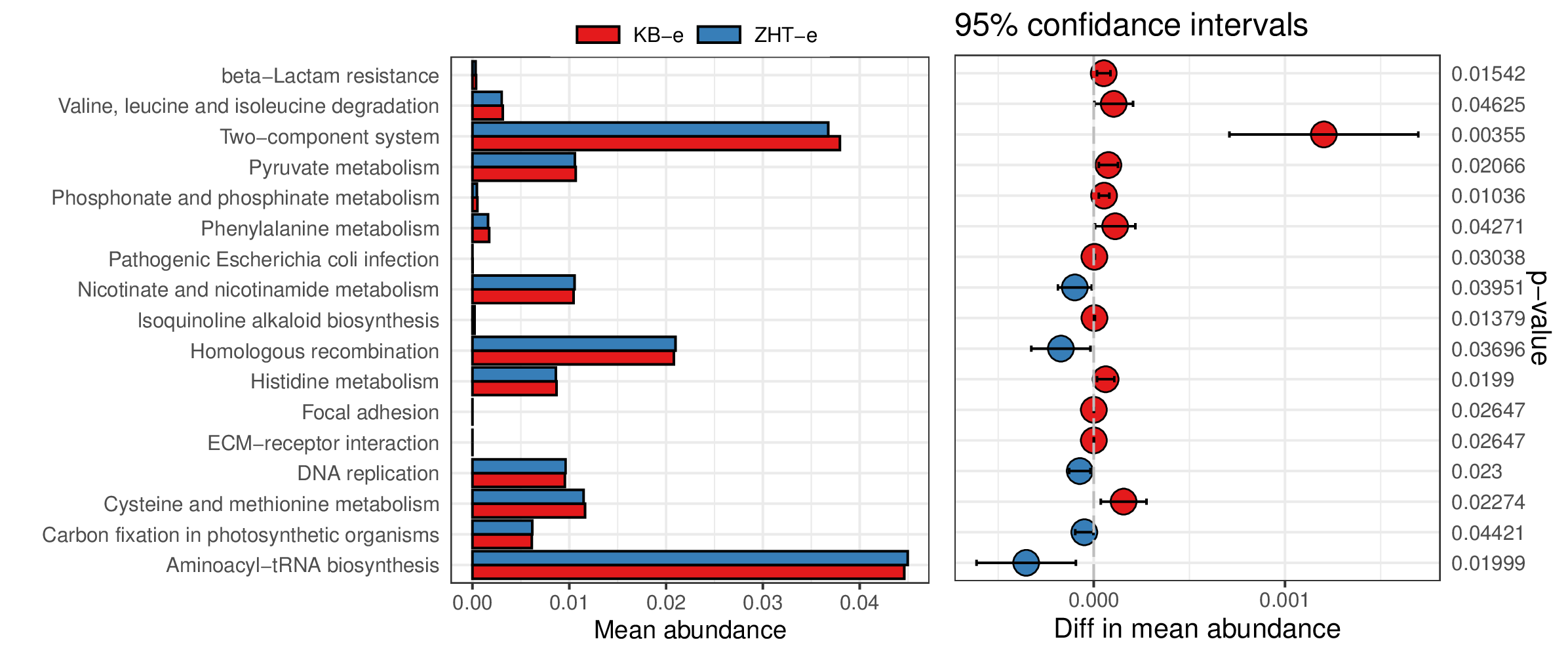

Supplement: Supplementary file 1 [file foods-12-04410-s001.zip › Supplementary Figure S2(b).png]
